# Therapeutic Mechanisms of Resmetirom and Semaglutide in MASLD/MASH: A Review of Inflammatory and Fibrotic Metabolites

**DOI:** 10.3390/metabo16080586

**Published:** 2026-08-18

**Authors:** Nobuyuki Toshikuni

**Affiliations:** Department of Gastroenterology and Hepatology, Kansai Medical University Medical Center, 10–15, Fumizono-cho, Moriguchi 570-8507, Japan; toshikuni.nob@kmu.ac.jp; Tel.: +81-6-6992-1001

**Keywords:** MASLD, MASH, metabolomics, therapeutic metabolomics, resmetirom, semaglutide, lipotoxicity, inflammation, fibrosis, precision pharmacotherapy

## Abstract

Metabolic dysfunction-associated steatotic liver disease (MASLD) is a leading cause of chronic liver disease, and its progressive inflammatory phenotype, metabolic dysfunction-associated steatohepatitis (MASH), is characterized by hepatocellular injury, inflammation, and fibrosis. These processes are closely linked to altered metabolite networks, including lipotoxic lipids, oxidized lipid mediators, bile acids, amino acids, acylcarnitines, redox-related metabolites, gut-derived metabolites, and extracellular matrix remodeling products. The recent introduction of resmetirom and semaglutide for MASH with moderate-to-advanced fibrosis provides two complementary models for interpreting these networks. Resmetirom, a liver-directed thyroid hormone receptor-β agonist, primarily enhances intrahepatic lipid handling, mitochondrial fatty acid metabolism, cholesterol turnover, and lipoprotein remodeling. Through this “inside-out” mechanism, resmetirom may reduce hepatocyte lipotoxic stress and secondarily attenuate inflammatory and fibrogenic signaling. Semaglutide, a glucagon-like peptide-1 receptor agonist, mainly acts through systemic metabolic unloading by reducing energy intake, body weight, insulin resistance, and adipose–liver substrate flux. Through this “outside-in” mechanism, semaglutide may improve the hepatic metabolite environment indirectly. However, histological improvement does not by itself establish causal metabolite mediators, and many specific metabolite-level mechanisms remain incompletely defined in human MASH. This review summarizes metabolite networks linked to inflammation and fibrosis in MASLD/MASH, compares the metabolic implications of resmetirom and semaglutide, and discusses how therapeutic metabolomics may support biomarker discovery, patient stratification, and precision pharmacotherapy.

## 1. Introduction

Metabolic dysfunction-associated steatotic liver disease (MASLD) is now one of the most prevalent chronic liver diseases worldwide and is strongly associated with obesity, type 2 diabetes mellitus, insulin resistance, dyslipidemia, and other cardiometabolic disorders [1,2,3,4]. Its progressive inflammatory phenotype, metabolic dysfunction-associated steatohepatitis (MASH), is characterized by steatosis, hepatocellular ballooning, lobular inflammation, and fibrosis [5,6]. Among these features, inflammation and fibrosis are the principal histological determinants of clinical outcome because they drive progression to cirrhosis, hepatic decompensation, hepatocellular carcinoma, and liver-related mortality [6,7,8].

The transition from steatosis to MASH reflects a shift from adaptive triglyceride storage to maladaptive metabolic stress. Increased free fatty acid delivery from insulin-resistant adipose tissue, hepatic de novo lipogenesis, mitochondrial overload, oxidative stress, altered bile acid signaling, gut–liver axis dysfunction, and extracellular matrix remodeling interact to create a pro-inflammatory and pro-fibrotic hepatic environment [6,9,10]. In this setting, metabolites are not merely biomarkers. Lipotoxic lipids, oxidized lipid products, eicosanoids, acylcarnitines, bile acids, amino acids, redox-related metabolites, gut-derived metabolites, and collagen-remodeling products may contribute to hepatocyte injury, Kupffer cell activation, hepatic stellate cell activation, and matrix deposition [9,10,11].

Until recently, treatment of MASLD/MASH relied mainly on lifestyle modification, weight reduction, and management of metabolic comorbidities. Sustained weight loss can improve steatosis, steatohepatitis, and fibrosis, but long-term maintenance is difficult in routine practice [12,13]. The introduction of pharmacological therapies for MASH with moderate-to-advanced fibrosis has therefore changed the field [14,15,16,17]. Resmetirom and semaglutide are especially informative because they improve MASH through distinct but convergent metabolic routes. Resmetirom is a liver-directed thyroid hormone receptor-β (THR-β) agonist that primarily targets intrahepatic lipid metabolism, mitochondrial fatty acid handling, cholesterol turnover, and lipoprotein remodeling [14,18]. It can be conceptualized as an “inside-out” model, in which improved hepatocyte metabolism may secondarily attenuate inflammatory and fibrogenic signaling. Semaglutide is a glucagon-like peptide-1 (GLP-1) receptor agonist that acts mainly through reduced energy intake, weight loss, improved insulin sensitivity, and decreased delivery of metabolic substrates to the liver [16,19,20]. It therefore represents an “outside-in” model of systemic metabolic unloading.

Importantly, these agents should not be discussed as treatments for all patients with MASLD/MASH. In the United States, the U.S. Food and Drug Administration (FDA) approved resmetirom, in conjunction with diet and exercise, for adults with noncirrhotic NASH/MASH and moderate-to-advanced fibrosis, consistent with fibrosis stages F2–F3, under an accelerated approval pathway based on improvement in steatohepatitis and fibrosis [14,15]. Continued approval may depend on verification of clinical benefit in confirmatory trials. The FDA also approved once-weekly semaglutide 2.4 mg injection for adults with noncirrhotic MASH and moderate-to-advanced fibrosis under accelerated approval, with ongoing assessment of whether histological improvements translate into reduced death, liver transplantation, and other liver-related events [16,17]. Therefore, the therapeutic mechanisms discussed in this review should be interpreted mainly in the context of noncirrhotic MASH with F2–F3 fibrosis, rather than MASLD/MASH in general.

This review examines MASLD/MASH pharmacotherapy through inflammatory and fibrotic metabolite networks. It first summarizes key pathways involved in hepatocellular injury, inflammation, and fibrosis; then, compares how resmetirom and semaglutide may modify these pathways; and finally, discusses evidence gaps and the future role of therapeutic metabolomics in response prediction and precision treatment.

For this narrative review, PubMed/MEDLINE and Google Scholar were searched from database inception, with the final search conducted in August 2026. Search terms included “MASLD,” “MASH,” “NASH,” “resmetirom,” “semaglutide,” “THR-β agonist,” “GLP-1 receptor agonist,” “metabolomics,” “lipidomics,” “ceramides,” “diacylglycerols,” “acylcarnitines,” “bile acids,” “redox,” “one-carbon metabolism,” “fibrosis,” “hepatic stellate cell,” “NLRP3,” “NF-κB,” “JNK,” “TGF-β,” “SMAD,” “YAP,” and “TAZ.” Priority was given to pivotal clinical trials, regulatory documents, human metabolomic or lipidomic studies, mechanistic studies relevant to MASH pathobiology, and recent reviews. Preclinical studies were included when they clarified mechanisms not yet fully established in treated human MASH. Because this was not a systematic review, formal risk-of-bias assessment and quantitative synthesis were not performed.

## 2. Metabolite Networks Linking Inflammation and Fibrosis in MASH

**Figure 1:**
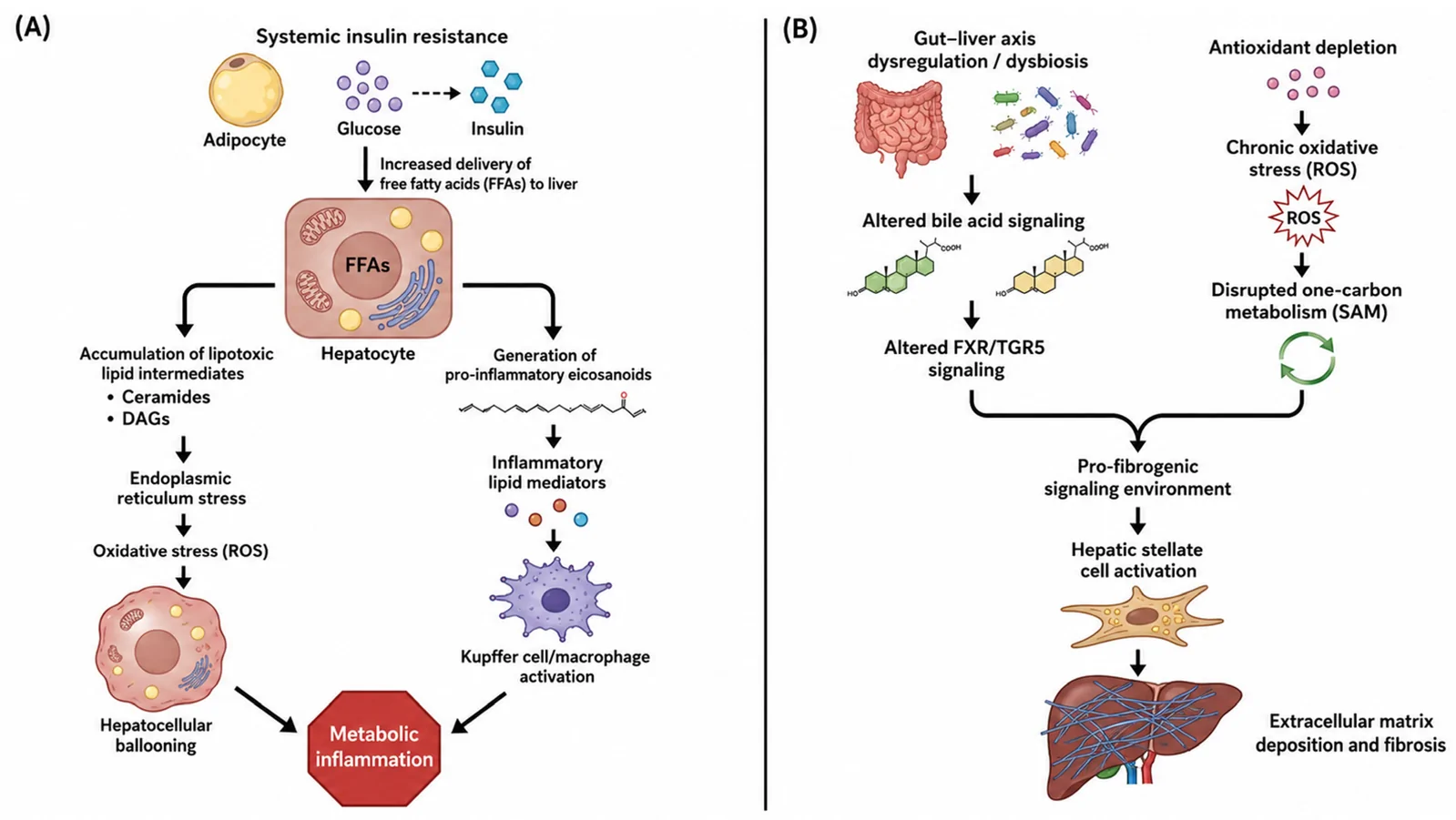
Metabolite networks linking metabolic overload to inflammation and fibrosis in metabolic dysfunction-associated steatohepatitis (MASH). (**A**) Systemic insulin resistance increases free fatty acid (FFA) delivery to the liver and promotes hepatic metabolic stress. When hepatic buffering capacity is exceeded, lipotoxic lipid intermediates, including ceramides and diacylglycerols (DAGs), accumulate and contribute to endoplasmic reticulum stress, oxidative stress, and the generation of pro-inflammatory eicosanoids and other inflammatory lipid mediators. These changes promote hepatocellular ballooning, Kupffer cell/macrophage activation, and metabolic inflammation. (**B**) In parallel, gut–liver axis dysregulation and dysbiosis alter bile acid signaling and downstream signaling through farnesoid X receptor (FXR) and Takeda G protein-coupled receptor 5 (TGR5). Antioxidant depletion and chronic oxidative stress, including reactive oxygen species (ROS) generation, together with disrupted one-carbon metabolism involving S-adenosylmethionine (SAM), create a pro-fibrogenic signaling environment that promotes hepatic stellate cell activation and extracellular matrix deposition, ultimately leading to fibrosis. Together, these interconnected pathways link metabolic overload to the inflammatory and fibrotic pathology of MASH. DAGs, diacylglycerols; FFAs, free fatty acids; FXR, farnesoid X receptor; MASH, metabolic dysfunction-associated steatohepatitis; ROS, reactive oxygen species; SAM, S-adenosylmethionine; TGR5, Takeda G protein-coupled receptor 5.

The progression from steatosis to MASH reflects failure of metabolic adaptation. In early MASLD, triglyceride storage may partly buffer excess lipid influx. When adipose tissue insulin resistance, hepatic de novo lipogenesis, impaired lipid export, and mitochondrial overload exceed hepatocellular buffering capacity, non-triglyceride lipid species and stress-associated metabolites accumulate. These metabolites connect systemic metabolic dysfunction to hepatocellular injury, immune activation, hepatic stellate cell activation, and extracellular matrix remodeling (Figure 1; Table 1) [6,9,10,21,22,23].

### 2.1. Lipotoxic and Inflammatory Metabolites

Excess delivery of free fatty acids (FFAs) from insulin-resistant adipose tissue is a central driver of hepatic metabolic stress. Under physiological conditions, insulin suppresses adipose tissue lipolysis; in insulin-resistant states, this suppression is impaired, increasing systemic and portal FFA flux to the liver [22]. Hepatocytes initially increase triglyceride synthesis, β-oxidation, lipid-droplet storage, and very-low-density lipoprotein export. When these pathways are overwhelmed, FFAs are redirected toward lipotoxic intermediates, including ceramides and diacylglycerols (DAGs) [21,22,23].

Ceramides and DAGs link lipid overload to insulin resistance, endoplasmic reticulum stress, mitochondrial dysfunction, oxidative stress, and inflammatory signaling [9,23,24]. Ceramides may impair insulin signaling and promote cellular stress or apoptosis, whereas DAGs can activate protein kinase C isoforms and worsen hepatic insulin resistance [9,23]. Free cholesterol is another lipotoxic mediator relevant to inflammation and fibrosis. Unlike esterified cholesterol, free cholesterol can accumulate in hepatocyte membranes, mitochondria, lysosomes, and hepatic stellate cells, where it may promote oxidative injury, macrophage activation, and sensitization of stellate cells to profibrotic stimuli [23,25]. These findings emphasize that MASH severity is determined not only by the amount of hepatic fat but also by lipid quality, localization, and biological activity [6,9,23].

Oxidized lipids, oxylipins, and eicosanoids further amplify the inflammatory metabolite environment. Lipid peroxidation products and oxidized phospholipids may act as danger-associated signals, while arachidonic acid metabolism generates prostaglandins, leukotrienes, hydroxyeicosatetraenoic acids, and related mediators that regulate Kupffer cell activation and inflammatory recruitment [6,9,23,24]. Thus, lipotoxic and oxidized lipid species provide a mechanistic bridge between lipid overload, hepatocyte injury, and lobular inflammation.

Lipotoxic stress also activates intracellular inflammatory pathways that connect hepatocyte injury to immune-cell recruitment. Saturated fatty acids, free cholesterol, oxidative stress, and organelle dysfunction can activate stress kinase and innate immune pathways, including c-Jun N-terminal kinase (JNK), nuclear factor-κB (NF-κB), and NLR family pyrin domain containing 3 (NLRP3) inflammasome signaling [6,9,26,27]. These pathways promote inflammatory mediator production, hepatocyte injury, macrophage activation, and progression from simple steatosis to steatohepatitis. However, in the context of resmetirom and semaglutide, these inflammatory pathways should be interpreted primarily as downstream targets of reduced metabolic stress rather than as proven direct pharmacological targets in human MASH.

### 2.2. Mitochondrial, Amino-Acid, and Redox Metabolites

Mitochondrial stress is another central feature of MASH. Increased β-oxidation may initially compensate for FFA overload, but persistent substrate excess can produce incomplete fatty acid oxidation, electron transport chain stress, and reactive oxygen species generation [6,28]. Acylcarnitines, intermediates of mitochondrial fatty acid transport and oxidation, have therefore been investigated as markers of mitochondrial overload or metabolic inflexibility, although their interpretation depends on chain length, fasting state, insulin resistance, and disease severity [29].

Redox-related metabolites connect mitochondrial stress to both inflammation and fibrosis. Excess lipid oxidation, lipid peroxidation, cytochrome P450 activation, and inflammatory cell activity increase oxidative stress, whereas glutathione, thioredoxin-related pathways, nicotinamide adenine dinucleotide phosphate availability, and other antioxidant systems maintain redox balance [6,29]. When these systems are depleted, lipid peroxidation, organelle injury, hepatocyte death, and inflammatory signaling are amplified. Because oxidative stress also promotes hepatic stellate cell activation, redox imbalance functions as a shared metabolic driver of inflammation and fibrogenesis [6,9,10].

Amino-acid and one-carbon pathways also link systemic metabolic dysfunction to hepatic injury and repair. Branched-chain and aromatic amino acids are often altered in obesity, type 2 diabetes, and MASLD/MASH, reflecting insulin resistance, impaired mitochondrial catabolism, and altered hepatic nitrogen handling [29]. Methionine, folate-cycle intermediates, choline, betaine, S-adenosylmethionine, and glutathione-related metabolites regulate methylation reactions, phosphatidylcholine synthesis, very-low-density lipoprotein export, antioxidant defense, and epigenetic control of inflammatory and fibrogenic genes [30,31].

### 2.3. Bile Acids, Gut-Derived Metabolites, and Fibrogenic Remodeling

Bile acids act as signaling molecules that regulate glucose metabolism, lipid metabolism, inflammation, gut barrier function, and fibrogenesis. Primary bile acids synthesized in the liver are modified by the gut microbiome into secondary bile acids, forming an enterohepatic signaling network mediated partly through farnesoid X receptor (FXR) and Takeda G protein-coupled receptor 5 (TGR5) [32]. In MASLD/MASH, altered bile acid composition, insulin resistance, hepatic inflammation, and gut dysbiosis may disrupt these pathways. Hydrophobic bile acid species may contribute to mitochondrial and endoplasmic reticulum stress, whereas impaired bile acid receptor signaling may reduce protective metabolic and anti-inflammatory responses [32].

Gut-derived metabolites, including short-chain fatty acids, indole derivatives, ethanol-related metabolites, trimethylamine-N-oxide-related pathways, and microbial products related to gut permeability, may either buffer or amplify liver injury [33]. Increased gut permeability can expose the liver to pathogen-associated molecular patterns, promoting Kupffer cell activation and inflammatory signaling. Conversely, beneficial microbial metabolites may support barrier integrity and immune regulation [33].

Fibrosis develops when chronic injury and inflammation sustain hepatic stellate cell activation and extracellular matrix deposition. Activated stellate cells require energy production, lipid remodeling, amino-acid utilization, and collagen synthesis [34,35]. Proline and hydroxyproline metabolism, collagen degradation products, oxidized lipids, free cholesterol, bile acid signaling, and extracellular matrix turnover markers are therefore closely linked to fibrotic remodeling [25,32,34,35,36]. Some of these metabolites may drive fibrogenesis, whereas others are biomarkers of matrix turnover or disease severity.

Fibrogenesis is also regulated by macrophage–stellate cell crosstalk and mechanotransduction. Persistent hepatocyte injury and macrophage-derived mediators activate hepatic stellate cells through pathways that include transforming growth factor-β (TGF-β)/SMAD signaling, osteopontin-associated signaling, and extracellular matrix remodeling [34,35,37,38,39]. As matrix accumulates, increasing tissue stiffness can further reinforce stellate cell activation through mechanosensitive pathways, including yes-associated protein/transcriptional coactivator with PDZ-binding motif (YAP/TAZ)-related signaling [37,38]. These pathways help explain how persistent metabolic injury becomes self-sustaining fibrotic remodeling, although their drug-specific modulation by resmetirom or semaglutide remains incompletely established in treated human MASH.

Taken together, inflammatory and fibrotic metabolite networks provide the biochemical context for interpreting resmetirom and semaglutide. Resmetirom primarily targets intrahepatic lipid handling, whereas semaglutide reduces systemic metabolic overload. These distinct therapeutic models may converge on reduced inflammatory injury and fibrogenic signaling.

## 3. Resmetirom: Liver-Directed Metabolic Remodeling

Resmetirom is a selective THR-β agonist developed to target hepatic metabolic dysfunction in MASH. THR-β is the predominant thyroid hormone receptor isoform in the liver and regulates fatty acid oxidation, mitochondrial activity, cholesterol turnover, bile acid synthesis, and lipoprotein metabolism [18,40]. Because impaired lipid disposal and lipotoxic stress are central to MASH progression, THR-β activation provides a rational liver-directed approach to modifying metabolic drivers of inflammation and fibrosis [6,9,23].

The clinical efficacy of resmetirom has been demonstrated in patients with biopsy-confirmed noncirrhotic NASH/MASH and moderate-to-advanced fibrosis. In the pivotal phase 3 MAESTRO-NASH trial, NASH resolution without worsening of fibrosis at week 52 occurred in 25.9% of patients receiving resmetirom 80 mg and 29.9% receiving resmetirom 100 mg, compared with 9.7% receiving placebo. Fibrosis improvement by at least one stage without worsening of disease activity occurred in 24.2% and 25.9% of the 80 mg and 100 mg groups, respectively, compared with 14.2% in the placebo group [14]. Resmetirom also reduced low-density lipoprotein cholesterol by 13.6% and 16.3% in the 80 mg and 100 mg groups, respectively, compared with a 0.1% change with placebo [14]. These results provide the clinical and lipid-profile basis for interpreting resmetirom as a liver-directed metabolic therapy.

**Figure 2:**
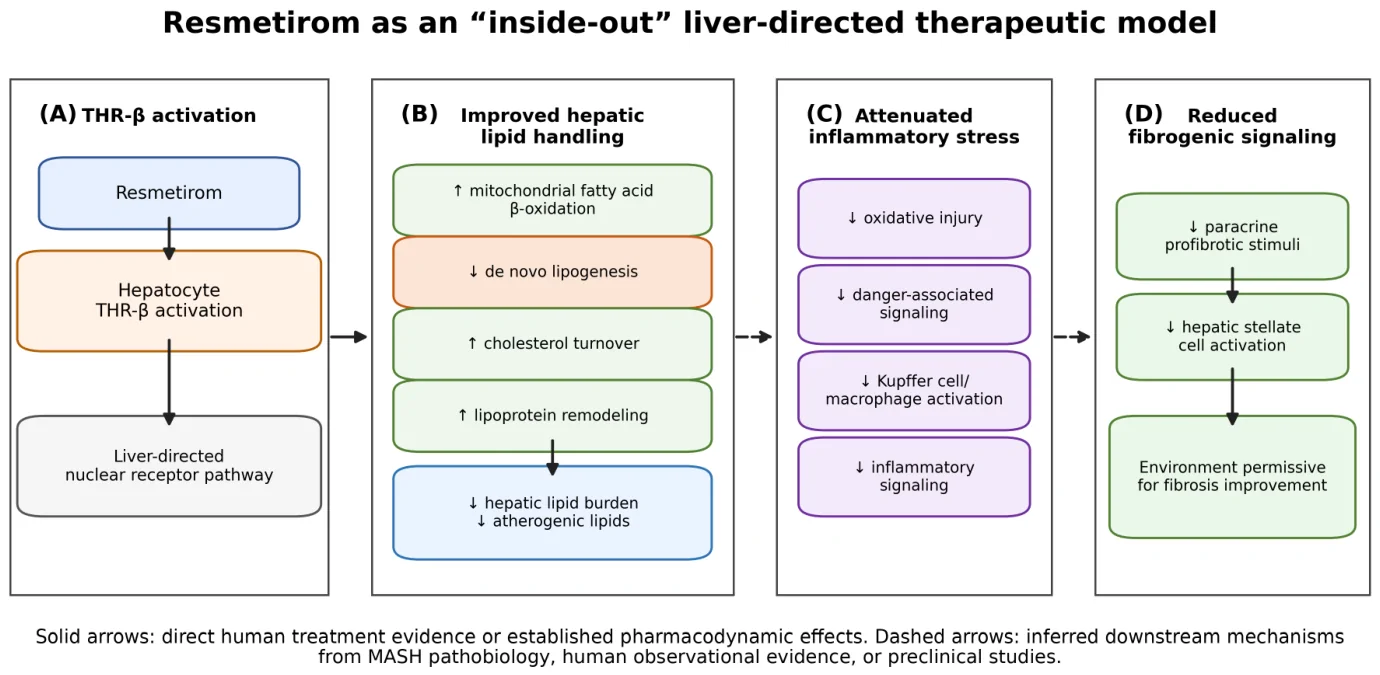
Resmetirom as an “inside-out” liver-directed therapeutic model. Solid arrows indicate direct human treatment evidence or established pharmacodynamic effects of resmetirom, including THR-β activation, improved hepatic lipid handling, liver fat reduction, and improvement in atherogenic lipid parameters. Dashed arrows indicate proposed downstream mechanisms inferred from MASH pathobiology, human observational evidence, or preclinical mechanistic studies. (**A**) Resmetirom activates hepatic thyroid hormone receptor-β (THR-β), a nuclear receptor pathway involved in hepatic lipid metabolism, mitochondrial activity, cholesterol turnover, bile acid synthesis, and lipoprotein remodeling. (**B**) Through THR-β activation, resmetirom improves intrahepatic lipid handling by enhancing mitochondrial fatty acid β-oxidation, reducing de novo lipogenesis, promoting cholesterol turnover, and improving lipoprotein remodeling. These effects reduce hepatic lipid burden, liver fat, and atherogenic lipid parameters. (**C**) Reduced hepatocyte lipotoxic stress may secondarily attenuate oxidative injury, danger-associated signaling, Kupffer cell/macrophage activation, and inflammatory signaling. (**D**) By reducing hepatocyte stress and inflammatory macrophage signaling, resmetirom may decrease the paracrine stimuli that maintain hepatic stellate cell activation and fibrogenic signaling, creating a hepatic environment permissive for fibrosis improvement. This model emphasizes that resmetirom acts primarily through liver-directed metabolic remodeling rather than direct immune suppression or direct inhibition of collagen synthesis. THR-β, thyroid hormone receptor-β. Up and down arrows indicate an increase/improvement and a decrease/reduction, respectively, in the indicated pathway or process.

Resmetirom can be viewed as an “inside-out” therapeutic model (Figure 2). Its primary action is not direct immune suppression or direct inhibition of collagen synthesis. Rather, resmetirom improves hepatocyte metabolic handling, thereby reducing intrahepatic stress signals that promote Kupffer cell activation, macrophage recruitment, hepatic stellate cell activation, and extracellular matrix remodeling [14,18,40,41]. Therefore, the metabolite-level consequences of resmetirom should be interpreted mainly as downstream effects of hepatic metabolic remodeling, unless directly demonstrated in treated human samples.

The most established metabolic effects of resmetirom are reductions in liver fat and improvements in circulating atherogenic lipid parameters [14,41]. These findings are consistent with THR-β-mediated enhancement of hepatic fatty acid oxidation, cholesterol clearance, bile acid-related metabolism, and lipoprotein remodeling [18,40]. From a metabolite-network perspective, these demonstrated effects support the hypothesis that resmetirom may reduce hepatic lipotoxic pressure. However, specific effects on ceramides, DAGs, oxylipins, acylcarnitines, bile acid species, redox metabolites, and one-carbon metabolites remain incompletely established in human MASH and should be regarded as inferred downstream mechanisms rather than proven treatment effects [9,21,22,23,24,42].

The anti-inflammatory and antifibrotic effects of resmetirom are therefore best described as metabolic de-escalation. Reduced hepatocyte lipid stress may decrease lipid peroxidation, oxidative injury, and danger-associated signals that activate Kupffer cells and recruit monocyte-derived macrophages [6,9,23,24]. Free cholesterol is especially relevant because experimental data suggest that it can sensitize hepatic stellate cells to profibrotic stimuli [25]. By decreasing hepatocyte stress and inflammatory macrophage signaling, resmetirom may reduce paracrine signals that maintain stellate cell activation. Candidate downstream pathways include TGF-β/SMAD signaling, osteopontin-associated pathways, collagen-related gene expression, and extracellular matrix turnover [34,35,39]. These mechanisms are biologically plausible but remain incompletely proven as causal pathways in treated human MASH.

Thus, resmetirom illustrates how targeting hepatocyte metabolic regulation can improve MASH resolution and fibrosis endpoints while also improving lipid profiles. Its strongest demonstrated effects are clinical histological endpoints, liver fat reduction, and atherogenic lipid improvement. Its proposed effects on specific inflammatory and fibrotic metabolite networks remain important hypotheses that require validation through paired lipidomic, metabolomic, and histological studies.

## 4. Semaglutide: Systemic Metabolic Unloading

**Figure 3:**
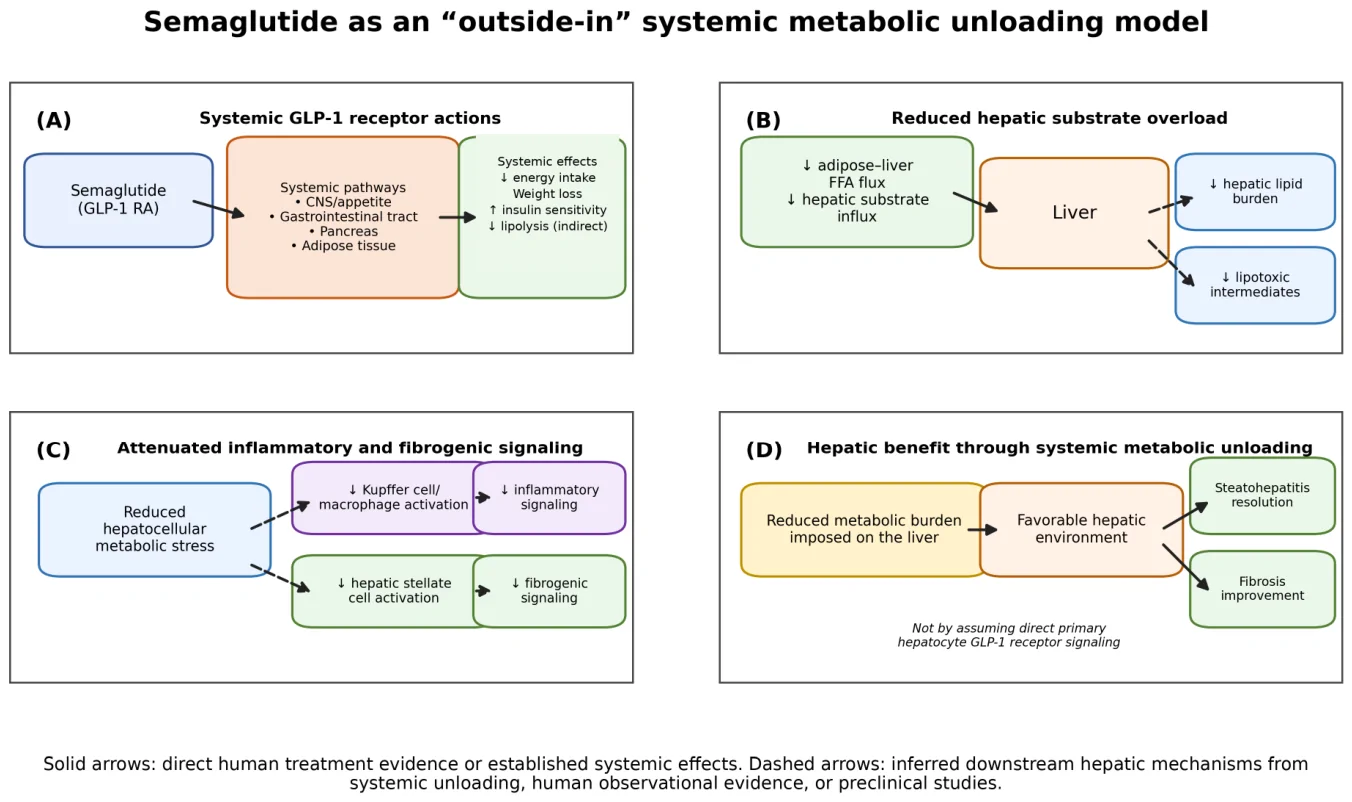
Semaglutide as an “outside-in” systemic metabolic unloading model. Solid arrows indicate direct human treatment evidence or established systemic effects of once-weekly semaglutide 2.4 mg, including reduced energy intake, weight loss, improved glycemic control, and biopsy-based efficacy endpoints. Dashed arrows indicate proposed downstream hepatic mechanisms inferred from systemic metabolic unloading, MASH pathobiology, human observational evidence, or preclinical mechanistic studies. (**A**) Semaglutide, a glucagon-like peptide-1 receptor agonist (GLP-1 RA), acts through systemic pathways, including the central nervous system, gastrointestinal tract, pancreas, and adipose tissue. These actions reduce energy intake, promote weight loss, improve glycemic control, and enhance insulin sensitivity. Reduced adipose–liver FFA flux is presented as a downstream consequence of reduced caloric intake, weight loss, and improved insulin action, rather than as proof of a direct adipocyte GLP-1 receptor-mediated mechanism. (**B**) These systemic effects decrease adipose–liver free fatty acid (FFA) flux and hepatic substrate influx, thereby reducing hepatic lipid burden and potentially reducing lipotoxic intermediates in hepatocytes. (**C**) Reduced hepatocellular metabolic stress may attenuate inflammatory signaling through Kupffer cells/macrophages and decrease fibrogenic signaling related to hepatic stellate cell activation. (**D**) Through systemic metabolic unloading, semaglutide may create a hepatic environment favorable to steatohepatitis resolution and fibrosis improvement. This model emphasizes that semaglutide acts mainly by reducing the metabolic burden imposed on the liver, rather than by assuming direct primary hepatocyte GLP-1 receptor signaling. FFA, free fatty acid; GLP-1 RA, glucagon-like peptide-1 receptor agonist. Up and down arrows indicate an increase/improvement and a decrease/reduction, respectively, in the indicated pathway or process.

Semaglutide is a long-acting GLP-1 receptor agonist that improves metabolic dysfunction through reduced energy intake, weight loss, improved glycemic control, enhanced insulin sensitivity, and cardiometabolic risk reduction [16,19,20,43]. In MASH, its therapeutic effect is best understood primarily as systemic metabolic unloading. Unlike resmetirom, which directly targets hepatic THR-β signaling, semaglutide reduces the upstream metabolic pressure imposed on the liver by nutrient excess, adipose tissue dysfunction, insulin resistance, and altered gut-derived signaling [19,20,42]. This “outside-in” model is summarized in Figure 3.

The pivotal human evidence comes from the phase 3 ESSENCE trial, which enrolled patients with biopsy-defined MASH and fibrosis stage 2 or 3 and evaluated once-weekly semaglutide 2.4 mg. At week 72, the two primary histological endpoints were reported as follows: steatohepatitis resolution without worsening of fibrosis occurred in 62.9% of patients receiving semaglutide compared with 34.3% receiving placebo, and fibrosis improvement without worsening of steatohepatitis occurred in 36.8% versus 22.4%, respectively. A combined secondary endpoint of steatohepatitis resolution and fibrosis improvement occurred in 32.7% versus 16.1%, respectively, and mean body weight decreased by 10.5% with semaglutide versus 2.0% with placebo [16]. These findings support the concept that systemic metabolic unloading can translate into biopsy-based benefit, while longer-term liver-related clinical outcomes remain under evaluation.

The strongest metabolite-related rationale for semaglutide is reduction in adipose–liver substrate flux. In obesity and insulin resistance, impaired suppression of adipose tissue lipolysis increases FFA delivery to the liver, promoting steatosis and lipotoxic injury [9,22]. By reducing energy intake, body weight, and insulin resistance, semaglutide may decrease adipose tissue lipolysis, hepatic substrate influx, and the metabolic drive for de novo lipogenesis [16,19,20]. Lower FFA delivery may then reduce the generation of ceramides, DAGs, oxidized lipids, and lipid peroxidation products, thereby decreasing endoplasmic reticulum stress, mitochondrial overload, oxidative injury, hepatocellular ballooning, and inflammatory signaling [9,21,22,23,24]. These downstream metabolite effects remain plausible but should not be interpreted as fully proven hepatic mechanisms without direct paired human metabolomic and histological evidence.

A separate consideration is the role of weight loss as a mediator. Semaglutide-induced weight loss can improve adipose tissue inflammation, adipokine balance, insulin sensitivity, hepatic fat content, and systemic substrate delivery [12,16,19,20,43]. Therefore, changes in circulating metabolites after semaglutide may reflect reduced caloric intake, altered diet composition, weight loss, adipose remodeling, improved glycemia, gut effects, or hepatic improvement. This distinction is essential because plasma metabolomics integrates signals from multiple organs and cannot by itself distinguish direct hepatic pathway modulation from systemic metabolic unloading [20,29].

Direct GLP-1 receptor-mediated hepatic effects should also be described cautiously. Semaglutide has clear systemic actions through central nervous system, gastrointestinal, pancreatic, and metabolic pathways. However, direct GLP-1 receptor expression and signaling in hepatocytes remain uncertain, and direct intrahepatic GLP-1 receptor-mediated mechanisms should not be assumed to explain MASH improvement [19,20]. Similarly, effects on adipose tissue biology may be mediated through weight loss, improved insulin action, and systemic metabolic changes rather than a single direct adipocyte mechanism.

The antifibrotic effect of semaglutide is likely mediated mainly by sustained reduction in metabolic injury. Fibrosis regression requires decreased hepatocyte injury, reduced inflammatory signaling, stellate cell deactivation, extracellular matrix degradation, and tissue remodeling [34,35]. Reduced lipotoxic pressure may decrease oxidized lipid formation, free cholesterol-associated injury, and macrophage–stellate cell crosstalk [23,24,25]. Improved redox balance and one-carbon metabolism may also influence fibrogenic responses [6,9,10,30,31]. The gut–liver axis is another possible pathway because GLP-1 receptor agonism alters gastric emptying, nutrient handling, body weight, and possibly gut microbiome composition, all of which may influence bile acid metabolism and microbial metabolite production [19,32,33]. These mechanisms remain best interpreted as plausible downstream effects unless supported by direct semaglutide-specific human evidence.

Overall, semaglutide illustrates the importance of extrahepatic metabolism in MASH. Its strongest demonstrated effects are weight loss, metabolic improvement, steatohepatitis resolution without worsening of fibrosis, and fibrosis improvement without worsening of steatohepatitis [16,43]. Its proposed effects on specific hepatic lipid, redox, bile acid, amino-acid, and fibrotic metabolite pathways should be interpreted as hypotheses generated from systemic metabolic unloading and MASH biology. Future studies should determine which circulating and hepatic metabolite signatures predict response, which are weight loss-dependent, and which reflect GLP-1 receptor-specific effects.

## 5. Comparative Interpretation, Evidence Gaps, and Future Directions

Resmetirom and semaglutide demonstrate two distinct but convergent therapeutic principles in MASH. Resmetirom enhances intrahepatic metabolic handling through THR-β activation, whereas semaglutide reduces systemic metabolic overload through GLP-1 receptor-mediated effects on energy intake, body weight, glycemic control, insulin sensitivity, and adipose–liver substrate flux. The former is best interpreted as liver-directed “inside-out” metabolic remodeling, and the latter as systemic “outside-in” metabolic unloading. Both approaches can reduce chronic metabolic stress that sustains hepatocellular injury, inflammation, and fibrogenesis, but they likely act through different upstream pathways [14,16,18,19,20,40,41,42].

A central issue is the distinction between demonstrated effects and inferred metabolite mechanisms. In human studies, resmetirom has demonstrated effects on MASH resolution without worsening of fibrosis, fibrosis improvement without worsening of disease activity, liver fat reduction, and atherogenic lipid parameters [14,41]. Semaglutide has demonstrated effects on steatohepatitis resolution without worsening of fibrosis, fibrosis improvement without worsening of steatohepatitis, body weight reduction, and systemic metabolic improvement [16,43]. By contrast, drug-induced changes in specific metabolite classes, including ceramides, DAGs, oxylipins, acylcarnitines, bile acid species, redox metabolites, one-carbon metabolites, and matrix-remodeling products, remain incompletely established in treated human MASH. Therefore, these metabolite pathways should be interpreted as biologically plausible downstream mechanisms unless directly supported by treatment-linked human metabolomic or lipidomic data. This evidence hierarchy is summarized in Table 2.

The major similarities and differences between resmetirom and semaglutide are summarized in Table 3. Resmetirom provides a model in which enhanced hepatic fatty acid oxidation, cholesterol handling, bile acid-related metabolism, and lipoprotein remodeling may reduce intrahepatic lipotoxic pressure. Semaglutide provides a model in which reduced energy intake, weight loss, improved insulin sensitivity, and decreased adipose–liver FFA flux may reduce hepatic substrate overload. These mechanisms are complementary but not equivalent. Resmetirom is more directly linked to hepatocyte metabolic regulation, whereas semaglutide modifies the hepatic environment mainly through systemic metabolic unloading.

Therapeutic metabolomics should therefore be designed to distinguish causal mediators from associated biomarkers. Metabolites associated with MASH may represent drivers of disease activity, adaptive responses, pharmacodynamic markers, or nonspecific indicators of disease severity. Plasma metabolomics is useful but has intrinsic limitations because circulating metabolites integrate signals from liver, adipose tissue, skeletal muscle, intestine, pancreas, kidney, immune cells, and dietary intake [20,29]. Future studies should integrate baseline and post-treatment histology with serial plasma metabolomics, hepatic lipidomics, bile acid profiling, microbiome analysis, transcriptomics, proteomics, imaging, and extracellular matrix turnover markers. Such designs would help determine whether metabolite changes precede biopsy response, differ between responders and nonresponders, and identify treatment-specific response signatures.

Patient heterogeneity is another important consideration. MASLD/MASH is shaped by obesity severity, type 2 diabetes, adipose tissue dysfunction, dyslipidemia, genetic risk, bile acid metabolism, microbiome composition, fibrosis stage, and cardiometabolic comorbidities [6,10,13,20]. A patient with dominant systemic substrate overload may respond differently from a patient with prominent hepatic lipid retention, dyslipidemia, or fibrotic remodeling. In the future, metabolite profiles may help identify patients most likely to benefit from liver-directed therapy, systemic weight-loss therapy, combination therapy, or earlier intervention before advanced fibrosis develops. At present, however, this precision approach remains investigational and requires prospective validation.

Emerging therapies will further test this framework. Dual and multi-receptor incretin agonists may produce deeper systemic metabolic unloading than GLP-1 receptor agonism alone [44]. Oral incretin-based agents may improve accessibility and adherence, although their role in MASH requires dedicated studies [45]. Fibroblast growth factor 21 analogs and other hepatic metabolic modulators may bridge liver-directed and systemic models by acting on adipose tissue, hepatic lipid metabolism, insulin sensitivity, and fibrosis-related pathways [46,47]. These agents should also be evaluated with the same evidence hierarchy, separating clinical efficacy, demonstrated metabolic changes, and inferred downstream mechanisms.

Combination or sequential therapy may eventually become important because MASH arises from both systemic metabolic overload and intrahepatic vulnerability. A liver-directed agent such as resmetirom and a systemic therapy such as semaglutide could theoretically target complementary components of the disease network. Nevertheless, combination therapy should be tested empirically rather than assumed to be additive. Metabolomics may help identify residual lipotoxic, inflammatory, or fibrogenic signatures after monotherapy and guide rational escalation.

Overall, resmetirom and semaglutide should be viewed not only as treatments but also as mechanistic probes. They show that MASH can be improved by enhancing intrahepatic metabolic capacity or by reducing systemic metabolic burden. The next challenge is to determine which metabolite pathways are causally linked to therapeutic response, which biomarkers predict benefit, and how these insights can be translated into precision pharmacotherapy.

## 6. Conclusions

MASLD/MASH develops through interconnected metabolite networks that link systemic metabolic dysfunction to hepatocellular injury, inflammation, and fibrosis. Lipotoxic lipids, oxidized lipid mediators, bile acids, amino acids, acylcarnitines, redox-related metabolites, gut-derived metabolites, and matrix remodeling products may act as mediators, modifiers, or biomarkers of disease progression [6,9,10,23,29,32,33,34,35,36].

Resmetirom and semaglutide provide two distinct therapeutic models for modifying these networks. Resmetirom acts mainly through liver-directed THR-β activation, improving hepatic lipid handling, mitochondrial metabolism, cholesterol turnover, and lipoprotein remodeling [14,18,40,41]. Semaglutide acts mainly through systemic metabolic unloading, reducing body weight, insulin resistance, and adipose–liver substrate delivery [16,19,20,43]. Both mechanisms can reduce metabolic stress and thereby attenuate inflammatory and fibrotic pathways.

Many specific metabolite-level mechanisms remain incompletely established in human MASH. Biopsy-based MASH resolution and fibrosis improvement confirm therapeutic efficacy but do not identify causal metabolite mediators [14,16]. Future studies integrating metabolomics, lipidomics, bile acid profiling, microbiome analysis, imaging, extracellular matrix biomarkers, and paired histology will be essential to define treatment-specific response signatures [20,29]. Such therapeutic metabolomics may ultimately guide patient selection, combination therapy and precision pharmacotherapy for MASLD/MASH.

## Figures and Tables

**Table 1:** Major metabolite networks linking inflammation and fibrosis in MASLD/MASH.

Metabolite Network	Representative Components	Relevance to Inflammation and Fibrosis
Lipotoxic lipid network	FFAs, ceramides, DAGs, free cholesterol	Promotes hepatocellular stress, insulin resistance, oxidative injury, Kupffer cell/macrophage activation, and stellate cell activation
Oxidized lipid and inflammatory mediator network	Lipid peroxidation products, oxidized phospholipids, oxylipins, eicosanoids	Links lipid overload to inflammatory signaling and immune-cell recruitment
Mitochondrial and redox network	Acylcarnitines, fatty acid oxidation intermediates, glutathione-related metabolites	Reflects mitochondrial overload, incomplete fatty acid oxidation, oxidative stress, and injury-driven fibrogenesis
Amino-acid and one-carbon network	Branched-chain amino acids, aromatic amino acids, methionine, choline, betaine, S-adenosylmethionine	Connects systemic insulin resistance, methylation capacity, antioxidant defense, and collagen-related repair responses
Bile acid and gut-derived metabolite network	Primary and secondary bile acids, short-chain fatty acids, indole derivatives, microbial products	Modulates gut–liver signaling, FXR/TGR5 pathways, barrier dysfunction, inflammation, and fibrogenesis
Matrix remodeling network	Proline, hydroxyproline, collagen degradation products, extracellular matrix turnover markers	Reflects extracellular matrix deposition, degradation, and fibrosis activity

FFAs, free fatty acids; DAGs, diacylglycerols; FXR, farnesoid X receptor; MASLD, metabolic dysfunction-associated steatotic liver disease; MASH, metabolic dysfunction-associated steatohepatitis; TGR5, Takeda G protein-coupled receptor 5.

**Table 2:** Human evidence and inferred metabolite mechanisms relevant to resmetirom and semaglutide.

Area	Demonstrated Human Evidence	Inferred Mechanisms/Evidence Gap
Disease-associated metabolomics/lipidomics	Human studies show altered lipidomic and metabolomic profiles associated with MASLD/MASH disease activity and fibrosis severity.	These findings provide biological context but do not prove drug-specific mechanisms.
Resmetirom	Improves MASH resolution without worsening fibrosis, fibrosis improvement without worsening disease activity, liver fat, and atherogenic lipid parameters.	Specific effects on ceramides, DAGs, oxylipins, acylcarnitines, bile acids, redox metabolites, and one-carbon metabolites remain incompletely established in treated human MASH.
Semaglutide	Improves steatohepatitis resolution without worsening of fibrosis, fibrosis improvement without worsening of steatohepatitis, body weight reduction, glycemic control, and systemic metabolic status.	Metabolite changes may reflect weight loss, altered diet, adipose remodeling, improved glycemia, gut effects, or hepatic changes rather than direct hepatic GLP-1 signaling.
Future therapeutic metabolomics	Serial human sampling with paired histology remains limited.	Integrated plasma metabolomics, hepatic lipidomics, bile acid profiling, microbiome data, imaging, extracellular matrix biomarkers, and histology are needed to identify causal mediators and response biomarkers.

DAGs, diacylglycerols; GLP-1, glucagon-like peptide-1; MASLD, metabolic dysfunction-associated steatotic liver disease; MASH, metabolic dysfunction-associated steatohepatitis.

**Table 3:** Comparison of resmetirom and semaglutide from a metabolite-network perspective.

Feature	Resmetirom	Semaglutide
Drug class/model	Selective THR-β agonist; liver-directed “inside-out” metabolic remodeling	GLP-1 receptor agonist; systemic “outside-in” metabolic unloading
Demonstrated principal effects	Improves MASH resolution, fibrosis improvement endpoint, liver fat, and atherogenic lipid parameters	Improves steatohepatitis resolution without worsening of fibrosis, fibrosis improvement without worsening of steatohepatitis, body weight, glycemic control, and systemic metabolic status
Main metabolic route	Enhances hepatic fatty acid oxidation, cholesterol turnover, bile acid-related metabolism, and lipoprotein remodeling	Reduces energy intake, body weight, insulin resistance, adipose–liver FFA flux, and hepatic substrate influx
Expected/hypothesized downstream effects	May reduce hepatic lipid burden, lipotoxic intermediates, cholesterol-associated stress, and downstream inflammatory signaling	May reduce adipose–liver FFA flux, hepatic lipid accumulation, lipotoxic stress, and systemic inflammatory input
Key uncertainty	Specific hepatic lipid and metabolite changes mediating biopsy response remain incompletely defined	Weight loss-dependent, GLP-1-specific, and liver-specific metabolite effects remain difficult to separate

FFA, free fatty acid; GLP-1, glucagon-like peptide-1; MASH, metabolic dysfunction-associated steatohepatitis; THR-β, thyroid hormone receptor-β.

## Data Availability

All data included in this manuscript come from publicly accessible results of published studies. No new data were created or analyzed in this study.

## Abbreviations

DAGs	Diacylglycerols
FFA(s)	Free fatty acid(s)
FXR	Farnesoid X receptor
GLP-1	Glucagon-like peptide-1
GLP-1 RA	Glucagon-like peptide-1 receptor agonist
JNK	c-Jun N-terminal kinase
MASLD	Metabolic dysfunction-associated steatotic liver disease
MASH	Metabolic dysfunction-associated steatohepatitis
NF-κB	Nuclear factor-κB
NLRP3	NLR family pyrin domain containing 3
ROS	Reactive oxygen species
SAM	S-adenosylmethionine
SMAD	Suppressor of mothers against decapentaplegic
TGF-β	Transforming growth factor-β
TGR5	Takeda G protein-coupled receptor 5
THR-β	Thyroid hormone receptor-β
YAP/TAZ	Yes-associated protein/transcriptional coactivator with PDZ-binding motif
